# Habitat Effects on the Breeding Performance of Three Forest-Dwelling Hawks

**DOI:** 10.1371/journal.pone.0137877

**Published:** 2015-09-30

**Authors:** Heidi Björklund, Jari Valkama, Erkki Tomppo, Toni Laaksonen

**Affiliations:** 1 The Zoology Unit, Finnish Museum of Natural History Luomus, University of Helsinki, Helsinki, Finland; 2 Department of Biosciences, University of Helsinki, Helsinki, Finland; 3 Natural Resources Institute Finland, Vantaa, Finland; 4 Department of Biology, University of Turku, Turku, Finland; INIBIOMA (Universidad Nacional del Comahue-CONICET), ARGENTINA

## Abstract

Habitat loss causes population declines, but the mechanisms are rarely known. In the European Boreal Zone, loss of old forest due to intensive forestry is suspected to cause declines in forest-dwelling raptors by reducing their breeding performance. We studied the boreal breeding habitat and habitat-associated breeding performance of the northern goshawk (*Accipiter gentilis*), common buzzard (*Buteo buteo*) and European honey buzzard (*Pernis apivorus*). We combined long-term Finnish bird-of-prey data with multi-source national forest inventory data at various distances (100–4000 m) around the hawk nests. We found that breeding success of the goshawk was best explained by the habitat within a 2000-m radius around the nests; breeding was more successful with increasing proportions of old spruce forest and water, and decreasing proportions of young thinning forest. None of the habitat variables affected significantly the breeding success of the common buzzard or the honey buzzard, or the brood size of any of the species. The amount of old spruce forest decreased both around goshawk and common buzzard nests and throughout southern Finland in 1992–2010. In contrast, the area of young forest increased in southern Finland but not around hawk nests. We emphasize the importance of studying habitats at several spatial and temporal scales to determine the relevant species-specific scale and to detect environmental changes. Further effort is needed to reconcile the socioeconomic and ecological functions of forests and habitat requirements of old forest specialists.

## Introduction

Unfavourable habitat changes are the main threats to threatened species worldwide [1]. Adverse anthropogenic habitat changes include habitat loss, deterioration and fragmentation, which can affect reproductive success and survival of species [2]. In addition to these direct impacts, habitat change can cause cascading effects among or between trophic levels through interspecific interactions [2,3]. For instance, increasing interspecific competition for high-quality habitats may force a subdominant competitor into inferior habitats [4]. Human-caused environmental change has thus the potential to affect species in different ways.

Changes in boreal forests caused by intensification of forestry practices since the 1960s are one of the greatest recent anthropogenic environmental changes in Northern Europe [5]. Regeneration cuttings, establishment of new forest and forest management have led to forest fragmentation, decreases in areas of old-growth forest, small openings and forest fires, and changes in forest age structure and tree species composition [5–9]. Structural changes in forests resulting from intensified forest management (e.g. even-aged stands, fewer large trees with thick branches and removal of decaying snags or malformed trees) have affected the quality of boreal forests as habitats and sites of reproduction for many taxa [10–12].

The consequences of changes in forests can be particularly dramatic for forest-dwelling birds of prey [13], because resources (food, nest sites) are usually sparse for top raptors [14] and furthermore, habitat change effects can escalate in food webs. Our study species, the northern goshawk (*Accipiter gentilis*, hereafter goshawk) with circumboreal distribution, and the common buzzard (*Buteo buteo*) and European honey buzzard (*Pernis apivorus*, hereafter honey buzzard) with Eurasian distributions, are capable of inhabiting diverse habitats, including coniferous, deciduous and mixed forests [15–22]. Mature forest and Norway spruce (*Picea abies*) seem important for these species [13,23–25]. Due to rather similar habitat requirements, the species can compete for territories and nest sites in their shared breeding range [26–29], although the goshawk is dominant as it can take over the nests of the other two species or even predate them [16,26,30].

Populations of the goshawk have declined in Northern Europe [31] and in parts of North America [20,25,32]. The common buzzard and the honey buzzard have overall stable populations except for their long-term declines in Northern Europe and a decline in the honey buzzard in Western Europe [31,33–35]. These declines can partly be due to intensified forest management and its consequences to prey availability [25,36–40]. The common buzzard and honey buzzard populations have decreased steeply in Finland and these species (but not the goshawk) are listed as vulnerable in the Finnish Red List [40]. The goshawk population has only slightly declined in Finland, which has raised concerns that the dominant goshawk could be replacing the buzzards from prime nest sites [26,39].

Despite the vast array of breeding habitat studies on these raptors, most of them are from the Temperate Zone. Only a few studies have been accomplished in the Boreo-nemoral Zone [21,23] and only one on the goshawk in the coniferous forest-dominated Boreal (Taiga) Zone in Europe [41]. This deficiency is striking, taking into account that these hawks have widespread distributions throughout the large Eurasian Boreal Zone [22]. Moreover, declining population trends of the hawks in Northern Europe have raised concerns of the state of the forests in their breeding grounds [36,40].

Breeding habitat studies are often conducted at rather small spatial scales (around nests, in restricted study areas), and typically over a short time period (such as a single year). It would be worthwhile to study habitat composition also at large scales, e.g. at a landscape scale of the nesting site, since these are often biologically more meaningful for species with large territories [25,36,42–45]. Additionally, it would be important to monitor temporal changes in habitat structure at these wider scales [25], and to plan management strategies in larger geographic areas for the benefit of hawks [25,46,47]. Modern methods, such as remote sensing combined with a geographic information system, can aid in fulfilling these needs.

We studied the European Boreal Zone breeding habitat and habitat-associated breeding performance of the goshawk, common buzzard and honey buzzard. Our work combines two unique datasets: geographically wide-scale and long-term Finnish hawk breeding data and the output data from a satellite image–aided multi-source national forest inventory (MS-NFI) at various distances around the nest trees. This allowed us to extend our research in several dimensions: from the nest-site level to the territory and landscape levels, from a local area to a nationwide geographic area and from few years to 19 years. Specifically, we aimed at investigating the following questions on multiple spatial scales: 1) is the breeding performance of the goshawk, common buzzard and honey buzzard associated with their breeding habitat in Northern European boreal forests, and 2) what are the typical characteristics of the boreal breeding habitats of the hawks, and have any temporal changes in the breeding habitats taken place during the study period that would reflect landscape changes. We were particularly interested in the role of old Norway spruce forest, since the importance of spruce and mature forest for the hawks has emerged from other studies, and forestry practices have particularly affected such forests [48].

## Materials and Methods

### Study area

Data on nests were included from the southern half of Finland (land area app. 154,000 km^2^) from an area extending approximately 640 km from south to north and 440 km from west to east (S1, S2 and S3 Figs). In general, the landscape is low-lying (mean height 152 m) and most of the land area is dominated by forests that are managed (77%). Forests in southern Finland are dominated by Scots pine (*Pinus sylvestris*, 56%), Norway spruce (31%) and broadleaved trees (11%, mainly silver birch *Betula pendula* and downy birch *B*. *pubescens*) [49].

### Study species

The goshawk, common buzzard and honey buzzard are middle-sized, forest-dwelling hawks [22,50] that build their stick nests under the crown layer [51]. Goshawks predate mainly forest grouse in Finland, but also other birds and mammals [37]. *Microtus* voles, forest grouse and hares are the main prey of common buzzards [52], and honey buzzards feed mainly on wasps Vespidae, but also on frogs and small birds [53]. Adult goshawks are sedentary while the common buzzard subspecies *B*. *b*. *buteo* (western Finland) is a short-distance migrant to central Europe [54]. The subspecies *B*. *b*. *vulpinus* (eastern Finland) and the honey buzzards are long-distance migrants to Africa [54].

### Nest data

Nest card data on hawk nests were gathered by volunteer raptor ringers and enthusiasts since 1982 as a part of the Finnish Common Birds-of-Prey Survey coordinated by the Finnish Museum of Natural History Luomus. A nest card includes information on nest location, nest type, breeding species and nest visits of the ringer [51]. Hawk territories can have several alternative nests which should be taken into account in statistical analyses to avoid pseudoreplication [55], since nests of the same territory have habitats potentially more similar than nests from different territories. Therefore, each nest was provided with a territory identity code (details in S1 Text).

We included nests with a verified breeding attempt that was ensured by observations of eggs, eggshells, chicks or remnants of chicks. This excludes occupied but only decorated nests, and possible breeding attempts in which eggs or chicks may have disappeared without trace. In total, we had data on the following numbers of breeding attempts: goshawk 1475 (from 861 nests), common buzzard 774 (529) and honey buzzard 166 (126).

For breeding performance analyses, we included breeding attempts with known results (S1, S2 and S3 Figs) and discarded nests that were not visited after the incubation or early nestling period. We quantified *breeding success* on a binary scale (successful, unsuccessful), and the breeding attempt was considered successful when at least one chick was raised to ringing age (14–28 days old). For *brood size* analyses, we included only those nests with an accurate number of chicks [51].

### Ethics Statement

Raptor ringers and enthusiasts followed the guidelines of Finnish Ringing Centre at the Finnish Museum of Natural History Luomus. According to these guidelines, unnecessary nest visits and nest climbing should be avoided during the breeding season in order to minimize disturbance. Ringing licences were issued by The Centres for Economic Development, Transport and the Environment. Hawk nests were located on different land types (private, state or company owned lands) which are accessible according to Finnish public right of access. Nests at protected areas were examined with a specific licence issued by The Centres for Economic Development, Transport and the Environment, or by Metsähallitus. No samples of protected species were taken for this study.

### Multi-source national forest inventory (MS-NFI) data

Our habitat data were based on MS-NFIs of the Natural Resources Institute Finland. The MS-NFI data is a combination of information from satellite images (Landsat Thematic Mapper, TM), field plots of Finnish national forest inventories (NFIs) and other georeferenced digital data [56,57], further details on the MS-NFI data in S2 Text.

We used the MS-NFI data (hereafter habitat data) from four MS-NFI periods (S2 Text) and we matched the year of habitat data (satellite image year) with the year of breeding data for each nest. Additionally, we generalized the habitat data for two preceding and two subsequent breeding years (but not before 1992, see S2 Text). For instance, if the habitat data around a nest were from the year 1999, these data were used for the breeding years 1997–2001 in this nest. As a result of the generalization of habitat data from four MS-NFI periods, we had breeding data on four breeding periods that we hereafter refer to as the first, second, third and fourth breeding periods. The *first breeding period* covered breeding years 1992–2004 (median breeding year of all species combined 1998), the *second breeding period* 2002–2007 (2004), the *third breeding period* 2005–2008 (2008) and the *fourth breeding period* 2008–2010 (2009). Since the number of honey buzzard nests was low in the fourth breeding period, we combined the third and fourth breeding periods as the *last breeding period* (median breeding year of all species 2008).

We retrieved habitat data in circles with radii of 100 m, 250 m, 500 m, 1000 m, 2000 m and 4000 m around each nest (see [41,45,58] for similar radii) that corresponded to areas of 3.14 ha, 19.6 ha, 78.5 ha, 314.1 ha, 1256.6 ha and 5026.4 ha, respectively. Habitat data from different scales ensured that we would include all potentially important habitat composition scales for each species.

Each pixel of the raw habitat data was classified into one of seven biologically relevant habitat classes (details in S2 Text): 1) spruce-dominated forest with tree stem volume ≥ 150 m^3^ ha^-1^ (hereafter old spruce forest); 2) other forest with tree stem volume ≥ 150 m^3^ ha^-1^ (hereafter other old forest, half of which consists of pine forest at all scales); 3) young thinning forest with tree stem volume ≥ 60 m^3^ –< 150 m^3^ ha^-1^ (young growing stock at the thinning cuttings stage [9]); 4) low stocking forest with tree stem volume from zero (treeless peatland or logged area) to < 60 m^3^ ha^-1^ (young seedling, seedling or seed tree stand, or peatland with low number of trees); 5) water; 6) arable land (of which cereals 52%, cultivated grasslands 29% and fallow areas 11%; [59]); and 7) built-up land (settlement, road or peat production area). We use here the terms ‘old spruce forest’ and ‘other old forest’ for brevity, but it should be noted that the correlation between tree age and size is not perfect [60,61]. By ‘old spruce forest’ or ‘other old forest’ we do not refer exclusively to natural, old-growth forest, which are rare in the study area [62]. However, our limit of tree stem volume ≥ 150 m^3^ ha^-1^ refers to advanced or mature forest, since the mean stock volume of advanced thinning stands is 163 m^3^ ha^-1^ and that of mature stands 207 m^3^ ha^-1^ in Finland (NFI 11: 2009–2012 [48]). We excluded clouds and areas without habitat data from the circular areas and then calculated the proportion of each habitat class in each area.

We used log-ratio transformation for the habitat variables in the study question 1 (method of [63] described in [64,65]). This was done to purge the mutual correlation of the habitat proportions because the sum of the habitat proportions is 1 (see S3 Text).

The habitat variable estimates within a radius contain some errors (error sources listed in [62], p. 91 onwards, see also S2 Text) that decrease when the size of the study area increases. Estimation of these errors and their incorporation in subsequent statistical analyses is a very complex and ambiguous issue [62,66], which we avoided in this study. Impacts of errors are potentially pronounced when evaluating temporal changes in habitat estimates (different model-based habitat data estimates); in such cases, comparisons can be done for a particular area, using NFI field data only [62].

### Statistical analyses

#### 1) How is breeding habitat associated with breeding performance?

We included data from the nests from all breeding periods for breeding success and brood size analyses. We investigated the influence of log-ratio habitat variables on breeding success and brood size with generalized linear mixed models (GLMMs) with the territory identity as a random effect. We preferred territory identity instead of nest identity as a random effect, because the landscape around alternative nests of the same territory is presumably similar and therefore the risk of pseudoreplication would still exist after considering nest identity as a random effect. The breeding success GLMMs of the goshawk and honey buzzard, assuming a binomial (with logit link) distribution, were then of the form (following [67]):

$$\begin{array}{l} Y_{ij}\sim Bin(1,p_{ij})\\ \text{log it}(p_{ij})=\alpha +\beta_{1}\times \text{log r}{\left({\text{old spruce forest}}_{r}\right)}_{ij}+\beta_{2}\times \text{log r}{\left({\text{young thinning forest}}_{r}\right)}_{ij}\\ +\beta_{3}\times \text{log r}{\left({\text{low stocking forest}}_{r}\right)}_{ij}+\beta_{4}\times \text{log r}{\left({\text{water}}_{r}\right)}_{ij}+\beta_{5}\times \text{log r}{\left({\text{arable land}}_{r}\right)}_{ij}\\ +\beta_{6}\times \text{log r}{\left({\text{built-up land}}_{r}\right)}_{ij}+a_{i}\\ a_{i}\sim N(0,\sigma_{a}^{2}) \end{array}$$ (1)

where *Y*_*ij*_ is 1 if nest j on territory i has a successful breeding attempt; otherwise *Y*_*ij*_ is 0. Logr is the log-ratio of the habitat variable and r is the radius. We assumed the random intercept *a*_*i*_ of the territory identity to be normally distributed (mean 0, variance $\sigma_{a}^{2}$). We found earlier with a longer-term dataset that the breeding success of the common buzzard decreases towards the north in Finland [51]. Thus, we added latitude to the breeding success model (1) of the common buzzard.

The brood size GLMMs assumed a Poisson (log link) distribution and included the same explanatory variables as in the model 1.

Since the appropriate scale was unknown, we first investigated, at which scale the habitat composition influences most the breeding performance of each species. Therefore, we fitted four GLMMs for both breeding success and brood size, using habitat variables within the radii of 100 m, 500 m, 1000 m and 2000 m. We standardized the model variables to mean = 0 and standard deviation (SD) = 0.5 [68]. We compared the Akaike information criterion (AIC) values of the models with habitat data at different scales, and chose for each species the model with the lowest AIC as the model best explaining breeding success or brood size [69]. If several models were almost equally good (AIC-difference to the best model ≤ 2 [68,69]), we inspected whether the parameter estimates of each of these top 2AIC models gave the same information than the best model. We further reduced the best model of each species if the log-ratio habitat variables were highly correlated (|r| > 0.7 [70]). We used the likelihood-ratio test to decide which of the correlated variables could be dropped. In cases where both variables could be dropped, we discarded the variable whose removal led to the lowest AIC. Model fit was evaluated graphically, and with parametric bootstrapping [71]. We tested the residuals of the best models for spatial autocorrelation with global Moran’s *I* [72] and found no spatial autocorrelation.

When fitting models for small counts (such as brood size of 1–5), but excluding zero (brood size 0) from possible values may potentially bias the parameter estimates of the model [67], while models including zero (brood sizes 0–5) could contain the same information already captured by breeding success models (since unsuccessful nests are the ones with brood size 0). To verify the brood size model results, we fitted zero-truncated generalized linear models (zero-truncated GLMs) with unstandardized log-ratio habitat variables, using the same variable sets as in the best models. The zero-truncation approach adjusts the parameter estimates by taking into account the exclusion of zero [67].

#### 2) Characteristics and changes in the boreal breeding habitat?

We included nests from the first and last breeding periods, and quantified first the breeding habitat proportions in the two periods at all scales (radii of 100–4000 m). For the analyses of changes in the breeding habitat, we chose the radii 100 m, 1000 m and 2000 m, where 100 m represents the nest-site scale, 1000 m the territory core scale and 2000 m the territory on a broad scale. The habitat proportions were arcsine square-root-transformed; this transformation is commonly used for proportions [73].

We analysed the differences in habitat proportions between the first and last breeding periods for each species with linear mixed-effect models, in which the dependent variable was an arcsine square-root-transformed habitat proportion, and the explanatory variable was the breeding period. We included territory identity as a random effect with a random intercept. If necessary, we allowed a different variance for the two breeding periods. We adjusted the threshold for a significant p-value with a Bonferroni correction.

We contrasted forest habitat changes around hawk nests with overall changes in forests in southern Finland using results of the NFI field data for the comparison [48]. The habitat classification in NFIs slightly differ from that of ours, but we used a classification that best matched with our habitat classes. We examined in the NFI data the changes in the area of > 60-year-old spruce-dominated forest (hereafter ‘older spruce-dominated forest’; this corresponds roughly to our old spruce forest), of > 60-year-old pine and deciduous forest (‘other older forest’, compares roughly with our other old forest), and of 21–60-year-old young forest (‘young forest’, corresponding approximately to our young thinning forest) in southern Finland between NFI-9 (1996–2003) and NFI-11 (2009–2012).

## Results

### 1) Association of breeding performance with the habitat

#### Breeding success

The overall breeding success was high in our nest card data. The proportion of successful breeding attempts was 89.8% for the goshawk (N = 1454 breeding attempts), 90.9% for the common buzzard (N = 762) and 87.0% for the honey buzzard (N = 161). Most breeding failures occurred at the egg-stage (S3 Table). Since nest cards may overestimate successful breeding attempts (early failures are missed or nest cards are filled more often from successful breeding attempts), we provide respective proportions of successful breeding attempts from the Raptor Questionnaire data of the Finnish Common Birds-of-Prey Survey in 1986–2014 for a comparison: 86.7% for the goshawk (N = 20928 breeding attempts), 88.7% for the common buzzard (N = 10111), and 80.9% for the honey buzzard (N = 2077, Finnish Museum of Natural History Luomus).

The breeding success of the goshawk was best explained by habitat proportions at the 2000 m radius scale. For the common buzzard, the best model was the one with habitat proportions within 100 m. The model with habitat proportions within 100 m was the best also for the honey buzzard, but habitat proportions at the 1000 m scale were almost as good in explaining honey buzzard breeding success.

**Fig 1:**
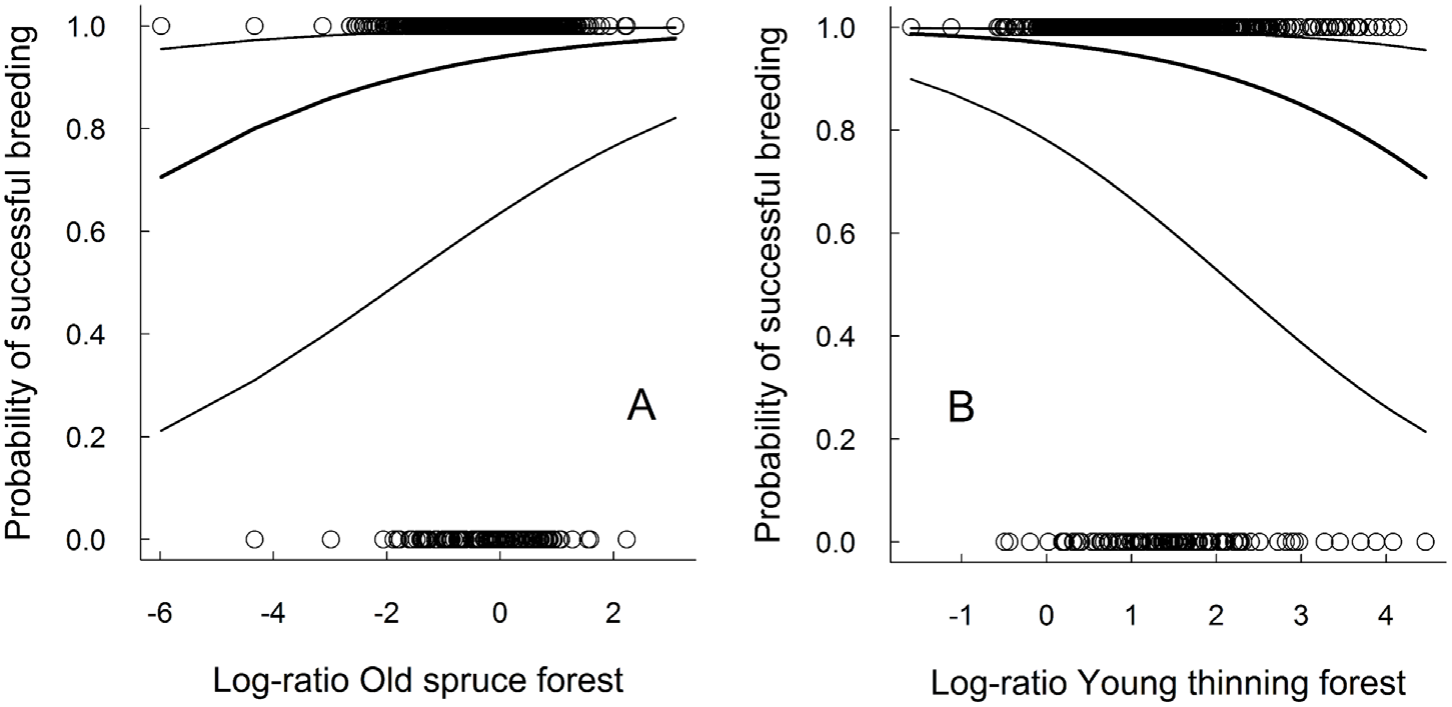
Probabilities of successful goshawk breeding based on the best breeding success GLMM (2000 m radius). All breeding attempts with a verified breeding result were included from all breeding periods. Thick line: predicted values, thin lines delineate 95% of the variation between territories in predicted values, dots: data points; 0 = unsuccessful, 1 = successful breeding attempt in the y-axis. (A) Probability of successful breeding along standardized log-ratio proportion of old spruce forest. (B) Probability of successful breeding along standardized log-ratio proportion of young thinning forest. Goshawk breeding success increases with (A) an increasing proportion of old spruce forest and (B) a decreasing proportion of young thinning forest within 2000 m around the nest.

The proportions of old spruce forest (Fig 1A) and water within 2000 m were significantly and positively associated with the breeding success of the goshawk, whereas the proportion of young thinning forest was significantly and negatively associated with goshawk breeding success (Fig 1B, Table 1). To illustrate the results on the biologically interesting original habitat proportion scale, we fitted additional breeding success GLMMs in which the only explanatory variable was each of the untransformed habitat variable proportion in turn at the 2000 m radius scale. Also here, the proportion of old spruce forest showed a significant positive and the proportion of young thinning forest a significant negative association with goshawk breeding success (S4 and S5 Figs). The proportion of built-up land had a significant positive association with goshawk breeding success, whereas the proportion of water and arable land were not significantly associated with goshawk breeding success.

**Table 1:** Logit estimates of the GLMMs^1^ that best explained the breeding success of each species. GLMMs included log-ratio habitat proportions within the radius of 100 m, 500 m, 1000 m or 2000 m, and the model with the lowest AIC was selected. Low stocking forest was removed from the best goshawk model due to high collinearity. Variance of the random variable (territory identity) describes variation among territories.

Species	Radius selected	Variable	Estimate	SE	z-value	p (>|z|)^2^
Goshawk	2000 m	Intercept	2.74	0.13	21.34	<0.001
		Old spruce forest	0.54	0.22	2.47	0.014*
		Young thinning forest	–0.93	0.27	–3.40	<0.001***
		Water	0.52	0.23	2.25	0.024*
		Arable land	0.22	0.25	0.90	0.369
		Built-up land	0.43	0.24	1.79	0.074
	random:	Territory, σ^2^:	1.25			
Common buzzard	100 m	Intercept	7.77	0.95	8.21	<0.001
		Old spruce forest	0.01	0.66	0.02	0.988
		Young thinning forest	0.63	2.11	0.30	0.767
		Low stocking forest	0.32	0.89	0.35	0.724
		Water	2.64	2.23	1.19	0.235
		Arable land	–1.55	1.23	–1.26	0.206
		Built-up land	0.13	1.19	0.11	0.912
		Latitude	–1.71	1.72	–0.99	0.322
	random:	Territory, σ^2^:	56.75			
Honey buzzard	100 m	Intercept	11.26	3.90	2.89	0.004
		Old spruce forest	0.37	7.67	0.05	0.961
		Young thinning forest	1.46	3.06	0.48	0.632
		Low stocking forest	–0.39	3.28	–0.12	0.905
		Water	5.86	6.31	0.93	0.353
		Arable land	0.68	11.02	0.06	0.951
		Built-up land	–1.75	3.59	–0.49	0.626
	random:	Territory, σ^2^:	288.95			

^1^GLMM: generalized linear mixed model,

^2^Significance levels:

*** <0.001,

* <0.05, n.s. ≥0.05

None of the habitat proportions of the best models were significantly associated with the breeding success of the common buzzard or honey buzzard (Table 1). We also inspected the second-best breeding success model for the honey buzzard (within 1000 m), but the interpretation was the same; none of the habitat variables were significantly associated with breeding success.

#### Brood size

The average brood size in successful nests was 2.89 (SD 0.86, N = 1167) for the goshawk, 2.26 (0.85, N = 592) for the common buzzard, and 1.79 (0.41, N = 121) for the honey buzzard. Two brood size models were included in the top 2AIC for both the goshawk (with habitat proportions within 2000 m and 1000 m) and common buzzard (within 2000 m and 500 m) while each of the four honey buzzard brood size models had a similar AIC (models with the lowest AIC in S1 Table). We verified the parameters of each competing model within the top 2AIC, and they made no change to the interpretation of the variables.

None of the habitat variables were significantly associated with the brood size for any of the hawks in the best models (S1 Table), or in the competing models. The results of the zero-truncated GLMs (not shown) were in general similar to those of the GLMMs, confirming the non-significant association of the habitat variables with the brood size.

### 2) Breeding habitat and habitat changes

**Fig 2:**
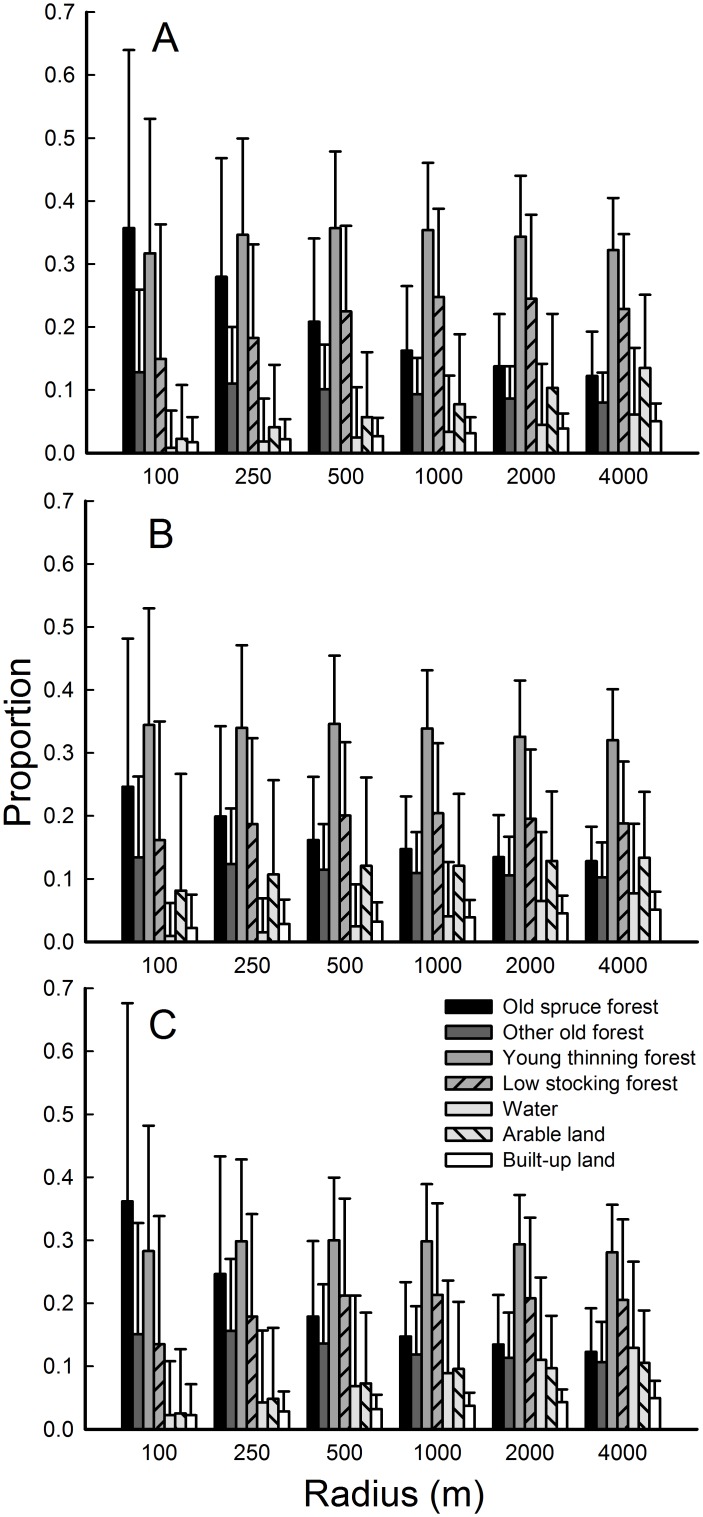
Habitat class proportions within different radii around nests. Proportions (mean, SD) of the seven habitat class estimates in the first breeding period. (A) The goshawk (N = 420 nests), (B) the common buzzard (N = 292), and (C) the honey buzzard (N = 76).

Habitat proportions at all scales around the nests are shown in Fig 2A–2C for the first breeding period, and in three scales for the first and last breeding periods (S2 Table).

The proportion of old spruce forest was highest for the goshawk at the nest-site scale (100 m) and decreased gradually with increasing radius (Fig 2A, S2 Table, see also S4 Text). The same pattern emerged for the honey buzzard, but in the first breeding period only (Fig 2C). In contrast, the proportions of water and arable land were low at the goshawk nest-sites and increased with increasing radius (Fig 2A). Young thinning forest was prominent in all radii and in both breeding periods for all species (Fig 2A–2C, S2 Table). The habitat proportions were very similar in different radii for the common buzzard (Fig 2B). However, arable land was a large component in common buzzard nest sites and at territory scales (Fig 2B), while a high proportion of water was apparent at the territory scales around honey buzzard nests (Fig 2C, S2 Table).

**Fig 3:**
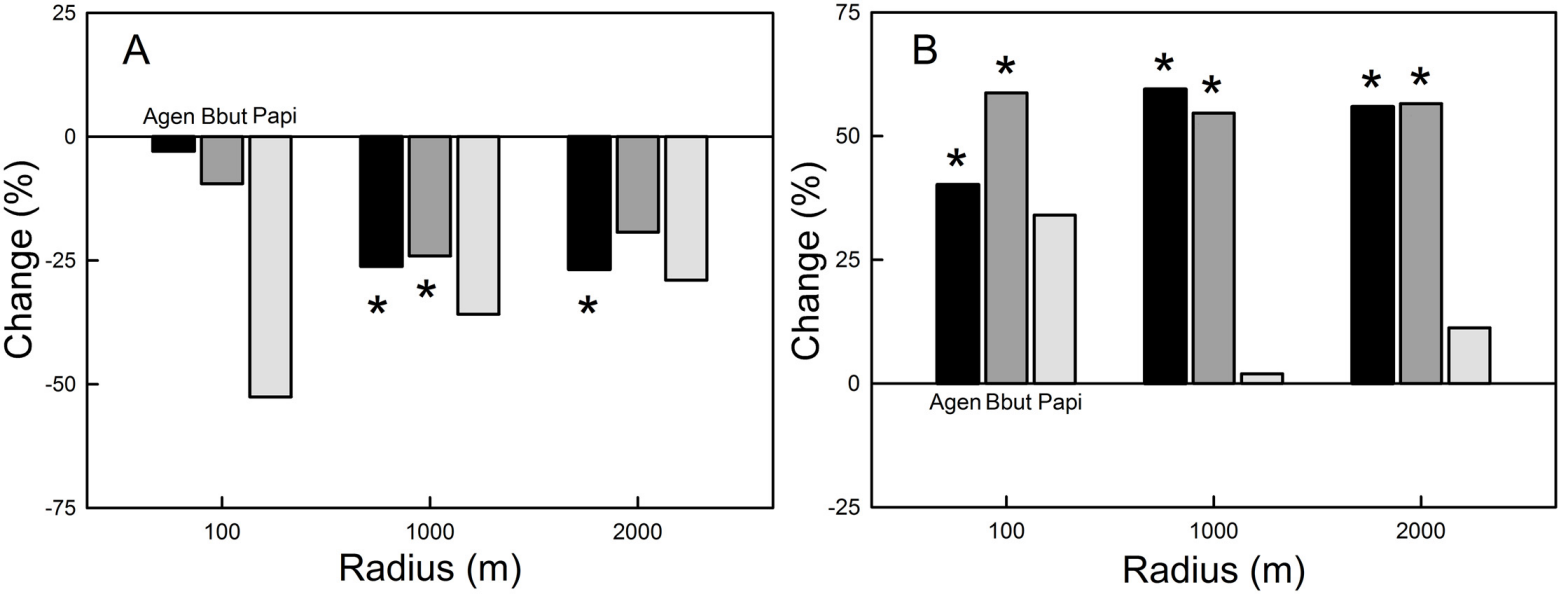
Changes (%) in proportions of (A) old spruce forest and (B) other old forest. Differences between the first and last breeding periods in arcsine square-root-transformed habitat proportions were tested within each radii for the Agen = goshawk, Bbut = common buzzard and Papi = honey buzzard with linear mixed-effect models (see S2 Table). Significant differences are indicated with asterisks. Number of nests (first / last breeding period): goshawk 420 / 269, common buzzard 292 / 137 and honey buzzard 76 / 19.

The proportion of old spruce forest declined significantly between the first and last breeding periods within 1000 m and 2000 m around goshawk nests, and within 1000 m around common buzzard nests (Fig 3A, S2 Table). At the nest-site scale (100 m), there was no significant difference in the proportion of old spruce forest between the first and last breeding periods for any of the species, although for the honey buzzard, the average proportion of old spruce forest was halved (Fig 3A). However, the sample size of the honey buzzard was low in the last breeding period.

The proportion of other old forest increased at all scales (100 m, 1000 m and 2000 m) for both the goshawk and the common buzzard (Fig 3B, S2 Table). Other significant breeding habitat changes for the goshawk included a decline in the proportion of low stocking forest and an increase in built-up land within 2000 m around the nests. No significant changes were detected in the proportions of young thinning forest, water and arable land between the breeding periods for any of the species.

The above-mentioned habitat changes were detected in territories that the hawks accepted for breeding and that can thus represent more suitable environments for the hawks than on average in the landscape. It was thus interesting to compare the habitat changes around nests to forest habitat changes throughout southern Finland. Areas of older spruce-dominated forest and other older forest decreased in southern Finland by 24% (from 19,040 km^2^ to 14,471 km^2^) and 10% (from 27,626 km^2^ to 24,838 km^2^), respectively. Instead, the area of young forest increased by 14% (from 42,901 km^2^ to 48,877 km^2^) [48].

## Discussion

We studied habitat effects on the breeding performance of three hawk species in boreal forest landscapes. The continuously declining population trends of these hawks have raised concerns about unfavourable changes in their breeding habitat, and some adverse changes were detected in this study. We found that goshawk breeding success increased with increasing proportions of old spruce forest and water, and decreasing proportions of young thinning forest within 2000 m around their nests. At the same time, old spruce forest decreased at the territory scales around goshawk nests and throughout southern Finland. We found no significant association with the habitat composition and the breeding success of the common buzzard and honey buzzard.

### Habitat-associated breeding performance

We found a preference of the goshawk for breeding in old spruce forest, since they predominated at goshawk nest sites (Fig 2A), while their proportion was much smaller on the landscape scale. Moreover, a higher proportion of old spruce forest around the nest increased goshawk breeding success. The goshawk’s preference for mature stands has been confirmed in many studies [13,17,21,24,25,37,44]. The goshawk favours large forest patches [42,74] and hunts inside the forest or at forest edges [37,75,76]. Furthermore, goshawk populations are sensitive to reductions in mature forest and prey populations [25,36]. The advantage of mature forest for the goshawk can operate through at least two pathways. First, forest grouse, the main prey of goshawks, have better breeding success when the proportion of old forest is higher in the landscape [77] and higher grouse populations benefit goshawks [78]. Secondly, open understorey space typical of mature stands allows for greater manoeuvrability when flying inside the forest [17,76] and therefore, a high proportion of old spruce forest in the territory is probably important for hunting grouse. Positive impact of high quality habitat (in terms of food availability) on breeding performance is known for the goshawk [79] and for other raptors [80]. Instead, young thinning forest proved to be disadvantageous for goshawk breeding success. Young forests are often dense and suboptimal for effective hunting, and goshawks appear to avoid them [75,76]. Better breeding success of the goshawk with increasing proportions of water at the territory scale can be due to improved foraging possibilities, since waterfowl are important alternative prey during the breeding season [81]. However, in contrast to the findings of Hargis et al. [46], the proportion of water was very low close to goshawk nest sites.

Habitat variables were not associated with the breeding success of the common buzzard and honey buzzard, or with the brood size of any of the hawks. While the goshawk has specific requirements for the nest tree and nest site, the buzzards may be in general less demanding with respect to breeding habitat ([13,16], but see [23]) or other factors are more important in regulating their reproduction. For example, common buzzard populations are known to be affected by food levels, weather, anthropogenic disturbance, intraspecific competition, and still to some extent by pesticides, such as rodenticides [82–86]. The honey buzzard has low productivity and it is sensitive to reductions in adult survival [33], which can result from environmental degradation or shooting in wintering, migration or breeding areas [33,87–90]. Also presence of predators (the goshawk or the eagle owl *Bubo bubo*) can explain the nest site choice and breeding performance of the buzzards [16,86,91]. These other factors thus seem to be more important than habitat in explaining the reproduction of the common buzzard and honey buzzard.

Alternatively, our delineation of habitat classes may have been inappropriate or they described insufficiently some specific landscape features that could be essential for the common buzzard and honey buzzard. For instance, López-López et al. [92] found that particular food resources dominated the space use of territorial raptors, and movements within territories occurred in specific directions so that territories were eccentric (nests were not in the centre of home ranges). A habitat composition approach based on circles around nests could thus miss important fine-scale features and misrepresent the spatial area used by the species. In search for food, raptors can also traverse distances beyond our largest radius [92]; for instance in Finland, satellite-tracked honey buzzard males were sometimes located over 10 km away from their nest (Patrik Byholm, unpublished data). Therefore, it can be that a general habitat composition approach is sufficient to determine high-quality breeding environments for some raptors (such as goshawk), but not for others (common buzzard, honey buzzard).

Nevertheless, it is still possible that habitat composition may have a role, impacting an earlier stage of common buzzard or honey buzzard breeding than what we measured. We analysed habitats around nests in which breeding was attempted. However, forests may have already lost some features that would be important for the settlement of these raptors, such as concealment of old forests [93] or prey species that have not been able to persist in the landscape. Consequently, if forests have changed a lot, the hawks do not necessarily settle in them at all. Hence, the landscape would support smaller populations of all these raptor species. Goshawks could then occupy the remaining suitable forests and potentially displace common buzzards and honey buzzards [29]. In such case, the proportion of forest habitat suitable for all species (old spruce forest, other old forest) should be increased in the landscape. However, it should be first analysed, how changes in habitat composition influence the occupancy and interactions of the species.

### Breeding habitat and habitat changes

Goshawks settled in forested areas far from water and arable land, which was shown by lower proportions of the latter at the nest-site scale than at large scales. The high proportion of arable land was striking for the common buzzard, which is in accordance with other studies. In the study areas of Kostrzewa [27] and Lõhmus [13], the common buzzard bred closer to woodland edges or arable land than the goshawk or the honey buzzard. The proximity of arable land may ensure an easy access to the habitat of prey, *Microtus* voles. The proportion of water was the highest for the honey buzzard at the territory scale. Amcoff et al. [23] found that honey buzzards tended to concentrate along luxuriant deciduous forest on fertile soils near lakes in Sweden, probably due to the abundant supply of small passerines in forests growing on nutrient-rich soils. The preference of the honey buzzard for nesting at sites of highest productivity was also confirmed by Selås [21] in Norway. In contrast, Lõhmus [13] described the honey buzzard in Estonia as the least demanding species in nest-site selectivity of six raptors, including the goshawk and common buzzard. Gamauf et al. [16] concluded that avoidance of goshawks dictated the nest-site selection of the honey buzzard. Breeding in habitats disfavoured by the goshawk (e.g. close to human settlement) could then be a tactic to reduce interference competition with the goshawk [16,27].

We found that old spruce forest declined during the 19 years at the territory scales around goshawk and common buzzard nests. Also the NFIs of southern Finland showed a similar trend. Our results thus indicate that the observed general decline in old forest and increase in younger stands since the 1960s in Finland [49] and throughout Fennoscandia [36] still continues. Since we analysed changes in habitat proportions, we cannot infer whether old spruce forest were lost as few large or several scattered patches. However, scattered logging is more probable, because most of the commercial forests are private owned (67% by area), and the average size of private owned forest is rather small, 30.3 ha [94]. Scattered logging leads to a fragmented landscape (in terms of mature forest) that seems to fulfil insufficiently the needs of the goshawk, preferring vast areas of mature forest. Although we found that goshawks have succeeded in retaining old spruce forest as their principal breeding habitat (no change in their proportion within 100 m around nests), the declining trend of the goshawk in Finland and in Fennoscandia [34,36] indicates that managed boreal forests support smaller goshawk populations.

The decrease in old spruce forest within territory scales around the goshawk and common buzzard nests was partly compensated for by an increase in other old forest, which contrasts with the general decreasing trend in other older forest, based on NFIs in southern Finland. However, other older forests were more prevalent and their decrease was smaller than that of older spruce-dominated forest in southern Finland NFIs. When old spruce forest decreased, goshawks and common buzzards evidently accepted other old forest in their territories rather than young forest (that were already common; Fig 2A–2C), since the NFIs showed that the area of young forest has increased in southern Finland, but not within territory scales around the hawk nests. Therefore, the discordance in trends of other old forest and other older forest can be explained by the fact that forest-dwelling hawks prefer mature forest instead of young forest. Even if the area of young forest increased, the hawks did not choose them proportionally to the increase in area.

Other old forest and young thinning forest consist mainly of Scots pine, the predominant tree species in forests in Finland, which has been favoured in forest regeneration [48]. Thus, young stands currently avoided by hawks host a growing body of pine forest that are later accepted in territories. Although the honey buzzard avoids breeding in mature pine forest [23], the goshawk seems to tolerate them and hunts in mature forest irrespective of tree species [76]. Since the amount of other old forest was not associated with the breeding success of the hawks, the increase detected in other old forest at the territory scales seems not to have had as yet any major adverse effects on the breeding performance of the hawks.

## Conclusions

Our results stress that it is essential to study the significance of habitat composition for breeding success at different scales in order to detect for each species the relevant scale. Analysing habitats at the nest-site scale seems to be insufficient for the goshawk, because we found that the territory scale affects breeding success the most. This is also in accordance with earlier findings that have confirmed the importance of habitats at large scales for the goshawk [43,76,95]. Landscape-scale habitat factors affect also the breeding success of forest grouse, the main prey of the goshawk [77]. Studies focusing only on the habitat composition in the proximity of nests could therefore miss the meaningful scale in terms of breeding success. The appropriate scale is dependent on the species and is not necessarily the same even for similarly sized competitors.

Our study in the Boreal Zone shows that adverse environmental changes can occur on a nationwide scale within a few decades. Most notably, a decrease of old forest continues and young forest become more prevalent in the landscape under intensive forest management. These trends likely have ongoing adverse consequences on many old forest species, since fragmentation and reduction in old forest lower their breeding performance and survival [77,93,96,97]. Moreover, young thinning forest was not preferred by any of the forest-dwelling hawks in our study, and a higher proportion of young thinning forest decreased the breeding success of the goshawk.

Our results for the goshawk and declines of old forest species further emphasize the importance to conserve old forests. There is thus an ongoing need to reconcile socioeconomic forest management objectives with the specific habitat requirements of declining forest-dwelling species, particularly of those depending upon old forest [98,99]. Foresters should ensure with forest management planning that enough suitable old forest for different species would be available on the landscape scale. For instance, the species could benefit from an ecosystem-based conservation strategy [100]. Wide-scale regional planning could ensure different-aged forests for the needs of forest-dwelling hawks and also old forest for the goshawk. However, as species differ in their responses to habitat changes, it is important to examine the effects of forestry from empirical data.

## Supporting Information

S1 Fig Distribution of goshawk breeding attempts. Light grey circles: successful breeding attempts (N = 1306); dark grey circles: unsuccessful breeding attempts (N = 148) from all breeding periods. We randomly added 0–1000 m to the nest coordinates in each breeding year to render visible the breeding attempts from the same nest in different years. Administrative borders: General map, National Land Survey of Finland, 2010.
(TIF)

S2 Fig Distribution of common buzzard breeding attempts. Light grey circles: successful breeding attempts (N = 693); dark grey circles: unsuccessful breeding attempts (N = 69) from all breeding periods. We randomly added 0–1000 m to the nest coordinates in each breeding year to render visible the breeding attempts from the same nest in different years. Administrative borders: General map, National Land Survey of Finland, 2010.
(TIF)

S3 Fig Distribution of honey buzzard breeding attempts. Light grey circles: successful breeding attempts (N = 140); dark grey circles: unsuccessful breeding attempts (N = 21) from all breeding periods. We randomly added 0–1000 m to the nest coordinates in each breeding year to render visible the breeding attempts from the same nest in different years. Administrative borders: General map, National Land Survey of Finland, 2010.
(TIF)

S4 Fig Goshawk breeding success along the proportion of old spruce forest. Probability of successful goshawk breeding based on a generalized linear mixed model, where the only explanatory variable was an untransformed proportion of old spruce forest at the 2000 m scale. Goshawk breeding success increases with an increasing proportion of old spruce forest. Thick line represents predicted values; thin lines delineate 95% of the variation between territories in predicted values, and dots are data points: 0 = unsuccessful, 1 = successful breeding attempts.
(TIFF)

S5 Fig Goshawk breeding success along the proportion of young thinning forest. Probability of successful goshawk breeding based on a generalized linear mixed model, where the only explanatory variable was an untransformed proportion of young thinning forest at the 2000 m scale. Goshawk breeding success decreases with an increasing proportion of young thinning forest. Thick line represents predicted values; thin lines delineate 95% of the variation between territories in predicted values, and dots are data points: 0 = unsuccessful, 1 = successful breeding attempts.
(TIFF)

S1 Table GLMMs that best explained the brood size of each species. (DOCX)

S2 Table Habitat class proportions and their comparisons between the breeding periods at three scales. (DOCX)

S3 Table Numbers of breeding attempts in different breeding results categories. (DOCX)

S1 Text Additional information on the Nest data. (DOCX)

S2 Text Multi-source national forest inventory (MS-NFI) data. (DOCX)

S3 Text Log-ratio transformation of habitat proportions. (DOCX)

S4 Text Characteristic of the boreal breeding habitat. (DOCX)
