# Supplementary figures and images for: Habitat Effects on the Breeding Performance of Three Forest-Dwelling Hawks
Source: PLoS One. 2015 Sep 30;10(9):e0137877. doi: 10.1371/journal.pone.0137877 (PMC4589344; doi:10.1371/journal.pone.0137877)

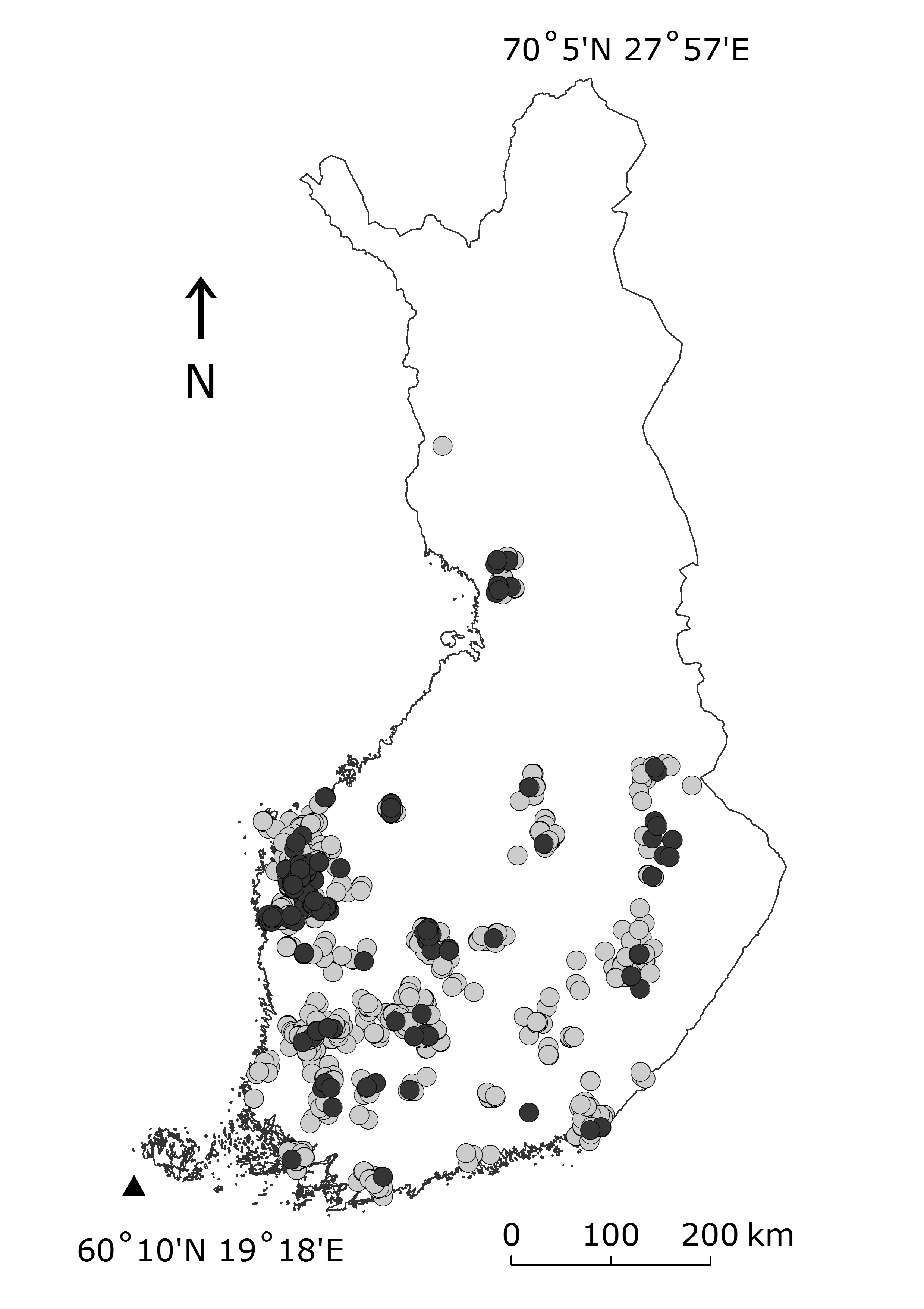

Supplement: S1 Fig — Light grey circles: successful breeding attempts (N = 1306); dark grey circles: unsuccessful breeding attempts (N = 148) from all breeding periods. We randomly added 0–1000 m to the nest coordinates in each breeding year to render visible the breeding attempts from the same nest in different years. Administrative borders: General map, National Land Survey of Finland, 2010. (TIF) [file pone.0137877.s001.tif]

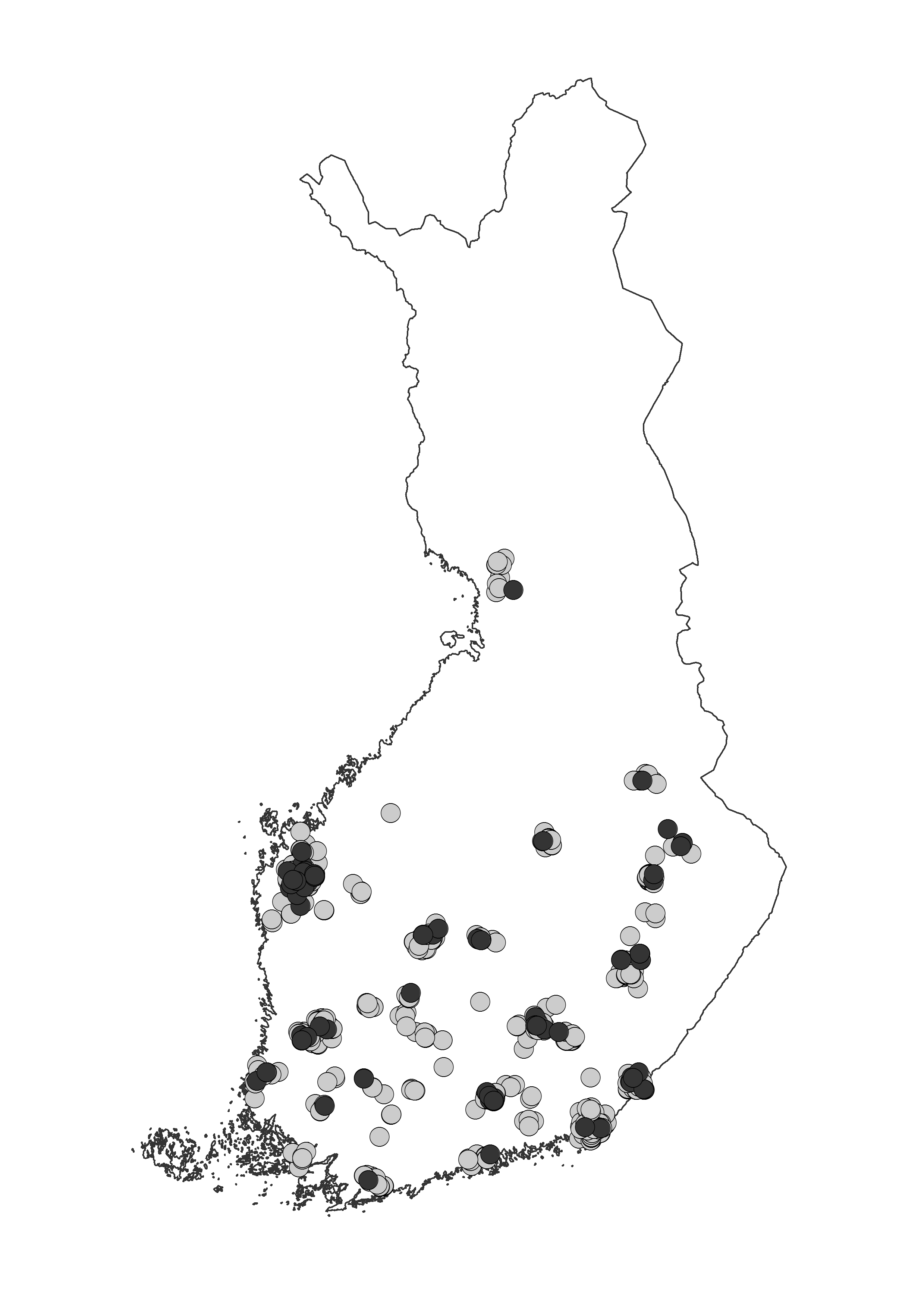

Supplement: S2 Fig — Light grey circles: successful breeding attempts (N = 693); dark grey circles: unsuccessful breeding attempts (N = 69) from all breeding periods. We randomly added 0–1000 m to the nest coordinates in each breeding year to render visible the breeding attempts from the same nest in different years. Administrative borders: General map, National Land Survey of Finland, 2010. (TIF) [file pone.0137877.s002.tif]

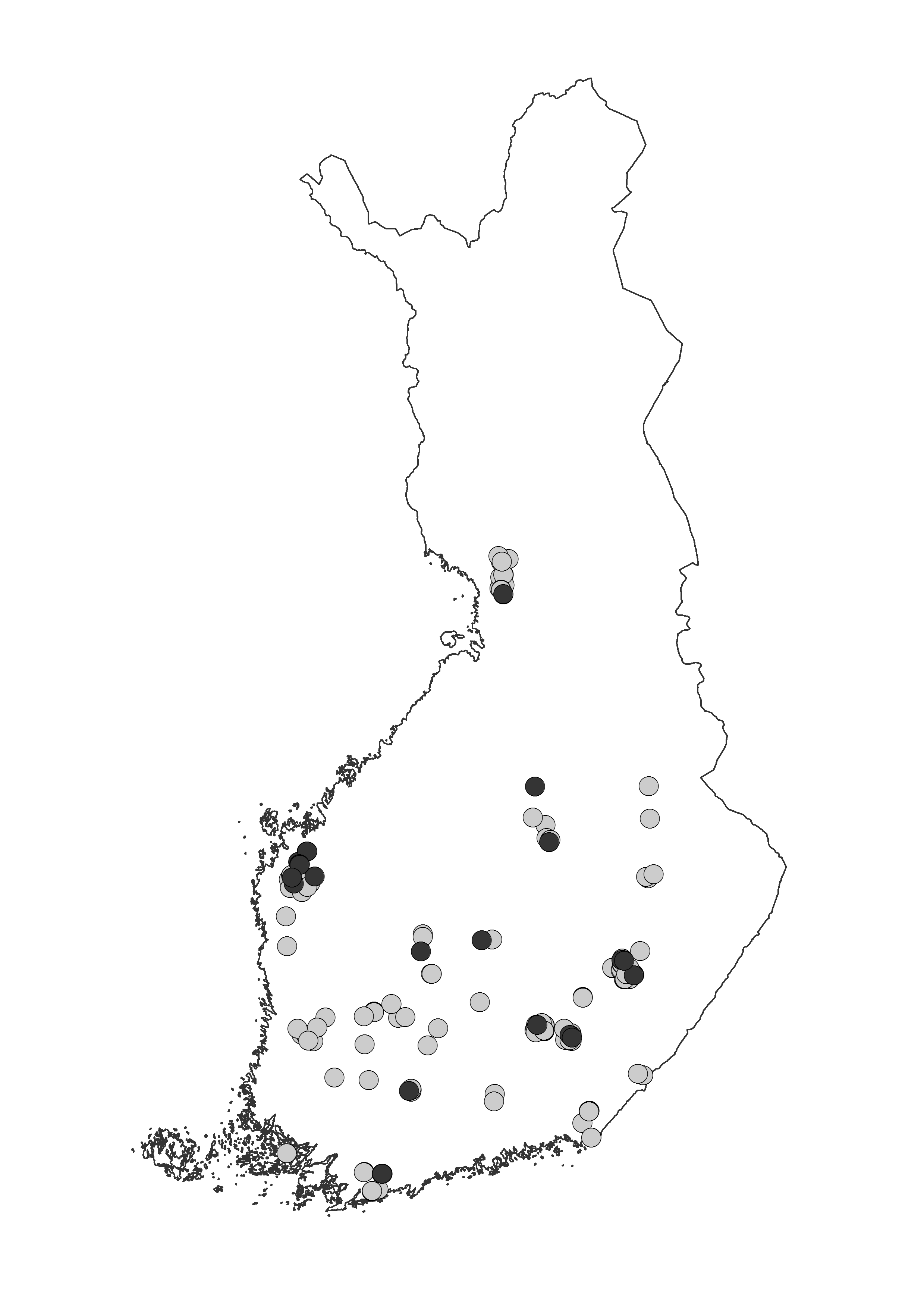

Supplement: S3 Fig — Light grey circles: successful breeding attempts (N = 140); dark grey circles: unsuccessful breeding attempts (N = 21) from all breeding periods. We randomly added 0–1000 m to the nest coordinates in each breeding year to render visible the breeding attempts from the same nest in different years. Administrative borders: General map, National Land Survey of Finland, 2010. (TIF) [file pone.0137877.s003.tif]

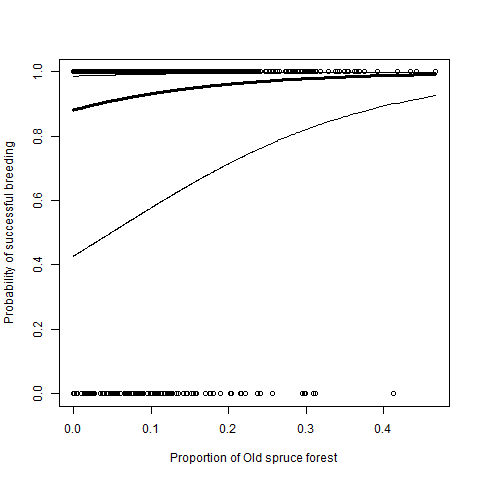

Supplement: S4 Fig — Probability of successful goshawk breeding based on a generalized linear mixed model, where the only explanatory variable was an untransformed proportion of old spruce forest at the 2000 m scale. Goshawk breeding success increases with an increasing proportion of old spruce forest. Thick line represents predicted values; thin lines delineate 95% of the variation between territories in predicted values, and dots are data points: 0 = unsuccessful, 1 = successful breeding attempts. (TIFF) [file pone.0137877.s004.tiff]

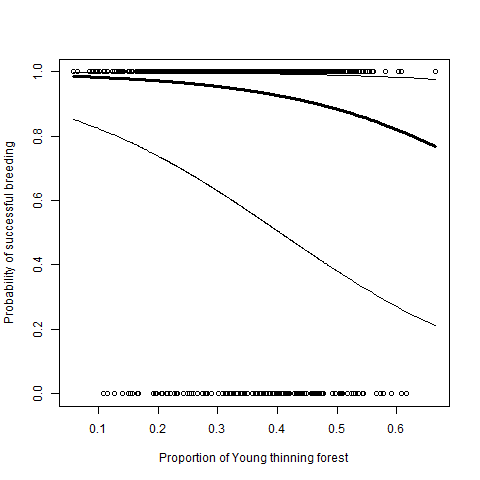

Supplement: S5 Fig — Probability of successful goshawk breeding based on a generalized linear mixed model, where the only explanatory variable was an untransformed proportion of young thinning forest at the 2000 m scale. Goshawk breeding success decreases with an increasing proportion of young thinning forest. Thick line represents predicted values; thin lines delineate 95% of the variation between territories in predicted values, and dots are data points: 0 = unsuccessful, 1 = successful breeding attempts. (TIFF) [file pone.0137877.s005.tiff]
