# Supplementary material for: Habitat Effects on the Breeding Performance of Three Forest-Dwelling Hawks
Source: PLoS One. 2015 Sep 30;10(9):e0137877. doi: 10.1371/journal.pone.0137877 (PMC4589344; doi:10.1371/journal.pone.0137877)
Supplement: S1 Table — (DOCX) [file pone.0137877.s006.docx]

**S1 Table**. Logit estimates of the GLMMs^1^ that best explained the brood size of each species. GLMMs included log-ratio habitat proportions within the radius of 100 m, 500 m, 1000 m or 2000 m, and the model with the lowest AIC was selected. Low stocking forest was removed from the best goshawk and common buzzard models due to high collinearity. Variance of the random variable (territory identity) describes variation among territories.

| Species | Radius selected | Variable | Estimate | SE | z-value | p (>\|z\|)^2^ |
| --- | --- | --- | --- | --- | --- | --- |
| Goshawk | 2000 m | Intercept | 1.06 | 0.02 | 61.40 | < 0.001 |
|  |  | Old spruce forest | 0.05 | 0.04 | 1.48 | 0.138 |
|  |  | Young thinning forest | 0.03 | 0.04 | 0.79 | 0.427 |
|  |  | Water | 0.01 | 0.04 | 0.26 | 0.796 |
|  |  | Arable land | 0.03 | 0.04 | 0.81 | 0.418 |
|  |  | Built-up land | –0.01 | 0.05 | –0.18 | 0.861 |
|  | random | Territory σ^2^: | 0 |  |  |  |
| Common buzzard | 2000 m | Intercept | 0.81 | 0.03 | 29.67 | < 0.001 |
|  |  | Old spruce forest | –0.08 | 0.06 | –1.19 | 0.233 |
|  |  | Young thinning forest | 0.10 | 0.07 | 1.49 | 0.136 |
|  |  | Water | –0.05 | 0.06 | –0.97 | 0.333 |
|  |  | Arable land | 0.04 | 0.07 | 0.64 | 0.521 |
|  |  | Built-up land | –0.07 | 0.07 | –0.98 | 0.327 |
|  | random | Territory σ^2^: | 0 |  |  |  |
| Honey buzzard | 100 m | Intercept | 0.58 | 0.07 | 8.47 | < 0.001 |
|  |  | Old spruce forest | –0.06 | 0.15 | –0.41 | 0.680 |
|  |  | Young thinning forest | 0.11 | 0.21 | 0.51 | 0.610 |
|  |  | Low stocking forest | –0.02 | 0.19 | –0.09 | 0.927 |
|  |  | Water | –0.06 | 0.17 | –0.37 | 0.712 |
|  |  | Arable land | 0.04 | 0.16 | 0.28 | 0.781 |
|  |  | Built-up land | –0.08 | 0.16 | –0.50 | 0.620 |
|  | random | Territory σ^2^: | 0 |  |  |  |

^1^GLMM: generalized linear mixed model, ^2^Significance levels: *** < 0.001, ** < 0.01, * < 0.05, n.s. ≥ 0.05
