# Supplementary material for: Habitat Effects on the Breeding Performance of Three Forest-Dwelling Hawks
Source: PLoS One. 2015 Sep 30;10(9):e0137877. doi: 10.1371/journal.pone.0137877 (PMC4589344; doi:10.1371/journal.pone.0137877)
Supplement: S2 Table — (DOCX) [file pone.0137877.s007.docx]

**S2 Table**. Habitat class proportions within 100 m, 1000 m and 2000 m around the nests of the three hawk species in the first and last breeding periods. All nests with eggs are included. First breeding period: 1992–2004; last breeding period: 2005–2010.

Habitat class proportions at all scales were compared between the first and last breeding periods with linear mixed-effect models (lme). The results are shown in lme columns on each ‘last’ row: asterisks = significant, n.s. = non-significant difference between the breeding periods. The threshold for a significant p-value was adjusted with a Bonferroni correction^1^. When necessary, different variances of habitat proportions were allowed for the two breeding periods.

|  | |  | | Proportion of the habitat class, mean, SD. Lme-significance^1^ indicates a difference between the breeding periods. | | | | | | | | | | | | | |  |
| --- | --- | --- | --- | --- | --- | --- | --- | --- | --- | --- | --- | --- | --- | --- | --- | --- | --- | --- |
| Species, | Breeding | | Old spruce | |  | Other old |  | Young |  | Low |  | Water |  | Arable |  | Built-up |  | |
| radius | period | | forest | |  | forest |  | thinning |  | stocking |  |  |  | land |  | land |  | |
|  | (nests) | |  | |  |  |  | forest |  | forest |  |  |  |  |  |  |  | |
|  |  | |  | | lme |  | lme |  | lme |  | lme |  | lme |  | lme |  | lme | |
| Northern |  | |  | |  |  |  |  |  |  |  |  |  |  |  |  |  | |
| goshawk |  | |  | |  |  |  |  |  |  |  |  |  |  |  |  |  | |
| 100 m | first (420) | | 0.36, 0.28 | |  | 0.13, 0.13 |  | 0.32, 0.21 |  | 0.15, 0.21 |  | 0.01, 0.06 |  | 0.02, 0.09 |  | 0.02, 0.04 |  | |
|  | last (269) | | 0.35, 0.27 | | n.s. | 0.18, 0.13 | *** | 0.31, 0.21 | n.s. | 0.12, 0.17 | n.s. | 0.02, 0.10 | n.s. | 0.01, 0.06 | n.s. | 0.02, 0.04 | n.s. | |
| 1000 m | first | | 0.16, 0.10 | |  | 0.09, 0.06 |  | 0.35, 0.11 |  | 0.25, 0.14 |  | 0.03, 0.09 |  | 0.08, 0.11 |  | 0.03, 0.03 |  | |
|  | last | | 0.12, 0.08 | | *** | 0.15, 0.07 | *** | 0.36, 0.10 | n.s. | 0.22, 0.12 | n.s. | 0.05, 0.11 | n.s. | 0.07, 0.10 | n.s. | 0.03, 0.04 | n.s. | |
| 2000 m | first | | 0.14, 0.08 | |  | 0.09, 0.05 |  | 0.34, 0.10 |  | 0.25, 0.13 |  | 0.04, 0.10 |  | 0.10, 0.12 |  | 0.04, 0.02 |  | |
|  | last | | 0.10, 0.07 | | *** | 0.13, 0.07 | *** | 0.35, 0.10 | n.s. | 0.22, 0.12 | *** | 0.07, 0.12 | n.s. | 0.09, 0.11 | n.s. | 0.04, 0.04 | *** | |
| Common |  | |  | |  |  |  |  |  |  |  |  |  |  |  |  |  | |
| buzzard |  | |  | |  |  |  |  |  |  |  |  |  |  |  |  |  | |
| 100 m | first (292) | | 0.25, 0.24 | |  | 0.13, 0.13 |  | 0.34, 0.19 |  | 0.16, 0.19 |  | 0.01, 0.05 |  | 0.08, 0.19 |  | 0.02, 0.05 |  | |
|  | last (137) | | 0.22, 0.22 | | n.s. | 0.21, 0.16 | *** | 0.29, 0.18 | n.s. | 0.11, 0.14 | n.s. | 0.01, 0.05 | n.s. | 0.14, 0.23 | n.s. | 0.02, 0.04 | n.s. | |
| 1000 m | first | | 0.15, 0.08 | |  | 0.11, 0.06 |  | 0.34, 0.09 |  | 0.20, 0.11 |  | 0.04, 0.09 |  | 0.12, 0.11 |  | 0.04, 0.03 |  | |
|  | last | | 0.11, 0.06 | | * | 0.17, 0.07 | *** | 0.33, 0.10 | n.s. | 0.19, 0.07 | n.s. | 0.03, 0.08 | n.s. | 0.13, 0.13 | n.s. | 0.04, 0.02 | n.s. | |
| 2000 m | first | | 0.13, 0.07 | |  | 0.11, 0.06 |  | 0.33, 0.09 |  | 0.20, 0.11 |  | 0.06, 0.11 |  | 0.13, 0.11 |  | 0.05, 0.03 |  | |
|  | last | | 0.11, 0.05 | | n.s. | 0.17, 0.06 | *** | 0.32, 0.09 | n.s. | 0.18, 0.07 | n.s. | 0.06, 0.08 | n.s. | 0.12, 0.10 | n.s. | 0.04, 0.02 | n.s. | |
| Honey |  | |  | |  |  |  |  |  |  |  |  |  |  |  |  |  | |
| buzzard |  | |  | |  |  |  |  |  |  |  |  |  |  |  |  |  | |
| 100 m | first (76) | | 0.36, 0.31 | |  | 0.15, 0.18 |  | 0.28, 0.20 |  | 0.14, 0.20 |  | 0.02, 0.09 |  | 0.03, 0.10 |  | 0.02, 0.05 |  | |
|  | last (19) | | 0.17, 0.22 | | n.s. | 0.20, 0.18 | n.s. | 0.31, 0.24 | n.s. | 0.11, 0.15 | n.s. | 0.11, 0.26 | n.s. | 0.07, 0.12 | n.s.**†** | 0.03, 0.06 | n.s. | |
| 1000 m | first | | 0.15, 0.09 | |  | 0.12, 0.08 |  | 0.30, 0.09 |  | 0.21, 0.15 |  | 0.09, 0.15 |  | 0.10, 0.11 |  | 0.04, 0.02 |  | |
|  | last | | 0.10, 0.08 | | n.s. | 0.12, 0.08 | n.s. | 0.29, 0.12 | n.s. | 0.20, 0.15 | n.s. | 0.11, 0.15 | n.s. | 0.14, 0.17 | n.s. | 0.04, 0.02 | n.s. | |
| 2000 m | first | | 0.13, 0.08 | |  | 0.11, 0.07 |  | 0.29, 0.08 |  | 0.21, 0.13 |  | 0.11, 0.13 |  | 0.10, 0.08 |  | 0.04, 0.02 |  | |
|  | last | | 0.10, 0.07 | | n.s. | 0.13, 0.08 | n.s. | 0.27, 0.08 | n.s. | 0.21, 0.15 | n.s. | 0.12, 0.13 | n.s. | 0.12, 0.13 | n.s. | 0.04, 0.02 | n.s. | |

^1^ Adjusted significance levels: *** < 0.000048, ** < 0.00048, * < 0.00238, n.s. ≥ 0.00238.

**†** Comparison made with Mann-Whitney U-test; linear mixed-effect model failed, due to low variation between the territories (small sample size).
