# Supplementary material for: Habitat Effects on the Breeding Performance of Three Forest-Dwelling Hawks
Source: PLoS One. 2015 Sep 30;10(9):e0137877. doi: 10.1371/journal.pone.0137877 (PMC4589344; doi:10.1371/journal.pone.0137877)
Supplement: S3 Table — (DOCX) [file pone.0137877.s008.docx]

**S3 Table**. Numbers of breeding attempts within different categories of verified breeding results. Breeding attempts were included from all four breeding periods.

| Result category | Northern goshawk | Common buzzard | Honey buzzard |
| --- | --- | --- | --- |
| Failed at egg-stage | 116 | 45 | 13 |
| Failed with small chicks | 31 | 22 | 4 |
| Failed with big young | 1 | 2 | 4 |
| Successful, chicks of ringing age | 961 | 490 | 100 |
| Successful, fledged young | 345 | 203 | 40 |
| **Sum** | **1454** | **762** | **161** |
