# Supplementary material for: Habitat Effects on the Breeding Performance of Three Forest-Dwelling Hawks
Source: PLoS One. 2015 Sep 30;10(9):e0137877. doi: 10.1371/journal.pone.0137877 (PMC4589344; doi:10.1371/journal.pone.0137877)
Supplement: S1 Text — (DOCX) [file pone.0137877.s009.docx]

**S1 Text.** **Additional information on the Nest data** (Materials and Methods).

Hawk territories can have several alternative nests, but the nest cards originally lacked a territory identity which should be taken into account in statistical analyses to avoid pseudoreplication [1]. Thus, we searched for and identified from the data alternative nests belonging to the same territory within a radius of 1000 m (for goshawk and honey buzzard) or 800 m (for common buzzard) around a nest. Thereafter, we extended the search with the same radius from each alternative nest found until no further nests were added to the territory. We chose these radii, based on the distances observed between nest sites elsewhere [2–4], and tried to avoid including nests from adjacent territories. We solved unclear cases based on nesting history details (e.g. the status of the nest in the previous year) from the nest cards, and finally provided each nest with a territory identity code.

Ringers were instructed to report data from one nest (usually the one with a breeding attempt) of a territory in each year to avoid pseudoreplication. However, if data from several alternative nests of a territory were reported within a year, we chose the alternative nest in which breeding had advanced the most in that year.

**References**

1. Hurlbert SH. Pseudoreplication and the design of ecological field experiments. Ecol Monogr. 1984;54: 187-211. doi: http://dx.doi.org/10.2307/1942661.

2. Penteriani V, Faivre B. Breeding density and landscape-level habitat selection of common buzzards (*Buteo buteo*) in a mountain area (Abruzzo Apennines, Italy). J Raptor Res. 1997;31: 208-212.

3. Hakkarainen H, Mykrä S, Kurki S, Tornberg R, Jungell S. Competitive interactions among raptors in boreal forests. Oecologia. 2004;141: 420-424. doi: 10.1007/s00442-004-1656-6.

4. Byholm P, Nikula A, Kenttä J, Taivalmäki J. Interactions between habitat heterogeneity and food affect reproductive output in a top predator. J Anim Ecol. 2007;76: 392-401. doi: 10.1111/j.1365-2656.2007.01211.x.
