# Supplementary material for: Habitat Effects on the Breeding Performance of Three Forest-Dwelling Hawks
Source: PLoS One. 2015 Sep 30;10(9):e0137877. doi: 10.1371/journal.pone.0137877 (PMC4589344; doi:10.1371/journal.pone.0137877)
Supplement: S2 Text — (DOCX) [file pone.0137877.s010.docx]

**S2 Text.** **Multi-source national forest inventory (MS-NFI) data** (Materials and Methods).

Multi-source national forest inventory (MS-NFI) combines satellite image data with appropriate national forest inventory (NFI) fieldwork data and additional georeferenced data to produce MS-NFI estimates. MS-NFI uses Landsat Thematic Mapper (TM) satellite images with the pixels resampled to a resolution of 20 m × 20 m.

Each pixel of the raw habitat data was classified into one of 27 predefined habitat classes which were further combined into seven biologically relevant habitat classes. Forest variables were predicted for all satellite image pixels. The nonparametric, improved k-nearest neighbour (ik-NN) method was employed [1,2]. During the first MS-NFI, satellite images were retrieved from various parts of Finland in different years [3]. Consequently, our first breeding period was longer. Later, the whole of Finland was processed essentially during one year, with some complementary images from adjacent years.

Since the fieldwork rotation of NFIs is longer than the period for satellite image acquisition, the years of the satellite image and of the respective NFI do not always match. However, the NFI field data were updated for extensive changes, such as regeneration cuttings, and currently also by means of growth models. We employed the year of the MS-NFI as a reference in generalizing the habitat data for the hawk breeding years.

Before generalization of the habitat data to the hawk breeding data, we took into account potential bias in the nest location, due to inaccurate coordinates. The nest coordinates pointed to the lower left corner of a 100 m × 100 m grid in the old Finnish National Coordinate System (KKJ). The nest can be located anywhere within this particular 1-ha grid cell. Thus, we added 50 m to both latitude and longitude to mark the central point of the grid cell as the nest location. This minimizes the potential bias between the coordinates and the true nest location.

We used the MS-NFI data (hereafter habitat data) from four MS-NFI periods: the first covered mainly the years 1992–1994 (with complementary satellite images up to 2002), the second the years 2004–2006, the third the years 2005–2007 and the fourth the years 2007–2009. We then generalized the habitat data from a specific year to the hawk breeding data of the same year as well as for ± two breeding years. However, due to the longer first MS-NFI period, generalization was not extended in breeding years before 1992 (which would have concerned only a few nests). If habitat data were available from more than one habitat period for a hawk breeding year (e.g. habitat data from 2007 (third period) or 2009 (fourth period) were available for a hawk nest with a breeding attempt in 2008), the earlier habitat data were preferred. It more probably described the real breeding habitat, because hawks still bred there. The *first breeding period* covered breeding years 1992–2004 (median breeding year of all species combined 1998), the *second breeding period* 2002–2007 (2004), the *third breeding period* 2005–2008 (2008) and the *fourth breeding period* 2008–2010 (2009). We further combined the third and fourth breeding periods as the *last breeding period* (median breeding year of all species 2008). The median breeding years were not necessarily the same as the main habitat data years, depending on how the habitat data were generalized and on the variation in the amount of hawk breeding data between years.

As mentioned in the section ‘Multi-source national forest inventory (MS-NFI) data’ in the text, habitat variable estimates within a radius may contain some unmeasurable errors due to sampling in the field, measurement and, particularly, estimation errors. Potential bias in errors of the habitat variable estimates can impact the accuracy of the habitat estimates, since unexplained variation shifts the estimates towards their means. Biased errors could especially affect the results of analyses on habitat change, since the true variation in habitat variable estimates can be higher. Our result for decreasing old spruce forest was congruent with the field data of the NFIs (see section ‘Breeding habitat and its changes’ in Results), while the trends in ‘other old forest’ (increase in hawk data) and ‘other older forest’ (decrease in NFI data) differed. The discordance can be explained by the fact that forest-dwelling hawks prefer mature forest instead of young forest.

**References**

1. Tomppo E, Halme M. Using coarse scale forest variables as ancillary information and weighting of variables in k-NN estimation: A genetic algorithm approach. Remote Sens Environ. 2004;92: 1-20. doi: dx.doi.org/10.1016/j.rse.2004.04.003.

2. Tomppo E, Haakana M, Katila M, Peräsaari J. Multi-source national forest inventory: Methods and applications. New York: Springer; 2008.

3. Tomppo E, Haakana M, Katila M, Mäkisara K, Peräsaari J. The multi-source national forest inventory of Finland – methods and results 2005. Working papers of the Finnish Forest Research Institute. 2009;111: 1-277. Accessible: www.metla.fi/julkaisut/workingpapers/2009/mwp111.pdf.
