# Supplementary material for: Habitat Effects on the Breeding Performance of Three Forest-Dwelling Hawks
Source: PLoS One. 2015 Sep 30;10(9):e0137877. doi: 10.1371/journal.pone.0137877 (PMC4589344; doi:10.1371/journal.pone.0137877)
Supplement: S3 Text — (DOCX) [file pone.0137877.s011.docx]

**S3 Text.** **The log-ratio transformation of habitat proportions** (Materials and Methods).

The sum of the habitat proportions is 1; they are thus mutually correlated, which renders the estimates of a model with all of them included unreliable. To purge this correlation, we used log-ratio transformation for the habitat variables in the first study question (method of Aitchison [1] described in [2,3]). With D habitat variables and x_i_ (i = 1,…, D) habitat proportions of the D variables, the log-ratio transformation is calculated as y_i_ = ln(x_i_/x_j_), where (i = 1,…, D, i ≠ j). Log-ratio transformation returns D–1 log-ratio habitat variables which can then be used in statistical procedures (the application is called compositional analysis). Since the denominator habitat proportion x_j_ is lost, x_j_ should preferably be unassociated with the dependent variable intended [3]. We chose ‘other old forest’ as the denominator habitat variable, because it is ubiquitous and yet includes a small proportion of every circular area. Furthermore, it was unrelated to dependent variable breeding success and brood size in univariate tests for each species.

Before log-ratio transformation, zero values in habitat proportions should be replaced with a value that is an order of magnitude less than the smallest value detected in the data [2,3]. We used 0.000001 as a replacing value.

Interpretation of log-ratios ln(x_i_/x_j_) involves how changes in x_i_ at the expense of x_j_ affect the dependent variable. Nevertheless, since use of the same denominator renders log-ratios mutually independent and our denominator was unassociated with the dependent variables, the effects of log-ratios can be interpreted as numerator effects only [3].

**References**

1. Aitchison J. The statistical analysis of compositional data. London: Chapman and Hall; 1986.

2. Aebischer NJ, Robertson PA, Kenward RE. Composition analysis of habitat use from animal radio-tracking data. Ecology. 1993;74: 1313-1325. doi: dx.doi.org/10.2307/1940062.

3. Hakkarainen H, Mykrä S, Kurki S, Korpimäki E, Nikula A, Koivunen V. Habitat composition as a determinant of reproductive success of Tengmalm's owls under fluctuating food conditions. Oikos. 2003;100: 162-171. doi: 10.1034/j.1600-0706.2003.11906.x.
