# Supplementary material for: Habitat Effects on the Breeding Performance of Three Forest-Dwelling Hawks
Source: PLoS One. 2015 Sep 30;10(9):e0137877. doi: 10.1371/journal.pone.0137877 (PMC4589344; doi:10.1371/journal.pone.0137877)
Supplement: S4 Text — (DOCX) [file pone.0137877.s012.docx]

**S4 Text. Characteristics of the boreal breeding habitat**

The goshawk, common buzzard and honey buzzard preferred the Norway spruce as a nest tree species. The proportion of nests in Norway spruce was 78% for the goshawk (N = 852 nests), 68% for the common buzzard (N = 524) and 93% for the honey buzzard (N = 122).

Nest sites and territory scales of the hawk species were dominated by forestry land (Table A below). In other Finnish studies on these hawks, the proportion of forestry land ranges from 73% of the study area [1] to 93% of a 1000 m circular area around goshawk nests [2], while the proportion of forestry land throughout Finland is 77% [3]. In Sweden, forestry land comprised 92% of Widén’s [4] goshawk study area.

**Table A.** Average proportions of forestry land in areas with different radii around the nests (data combined of nests from the first and last period). Forestry land includes old spruce forest, other old forest, young thinning forest and low stocking forest.

|  | Proportion in % (SD) | | |
| --- | --- | --- | --- |
| Radius (m) | Goshawk (689 nests) | Common buzzard (429) | Honey buzzard (95) |
| 100 | 95 (11) | 87 (21) | 90 (18) |
| 1000 | 85 (14) | 80 (14) | 76 (17) |
| 2000 | 81 (15) | 77 (14) | 74 (14) |
| 4000 | 75 (15) | 74 (13) | 72 (13) |

We further examined the proportions of old coniferous and deciduous forest in the hawk nest sites and at territory scales. Our habitat class “other old forest” is composed of old pine-dominated forest, old deciduous forest and old mixed forest. We separated the old pine-dominated forest and combined them with our habitat class old spruce forest to create a habitat class *old coniferous forest*. Respectively, we separated the *old deciduous forest* as another habitat class (Table B below).

**Table B.** Average proportions of old coniferous and old deciduous forest at two scales around hawk nests. Nests from the first breeding period were included.

|  |  | Proportion % (SD) | | |
| --- | --- | --- | --- | --- |
| Habitat | Radius (m) | Goshawk | Common buzzard | Honey buzzard |
|  |  | (420 nests) | (292) | (76) |
| Old coniferous forest^1^ | 100 | 42.2 (27.9) | 31.3 (24.2) | 44.2 (30.7) |
|  | 2000 | 18.0 (8.9) | 19.2 (7.5) | 19.4 (8.8) |
| Old deciduous forest^2^ | 100 | 0.9 (2.6) | 1.4 (3.5) | 1.0 (2.3) |
|  | 2000 | 0.8 (0.8) | 0.9 (0.9) | 1.1 (1.0) |

^1^ Old spruce forest + old pine forest; the latter is a fraction of the habitat class ‘other old forest’

^2^ Another fraction of the habitat class ‘other old forest’

Old coniferous forest predominated at the nest sites of the hawks, while old deciduous forest constituted only a small proportion in nest sites and at large scales (Table B). This reflects the overall predominance of conifers in boreal forests. Deciduous trees are more common at the nest sites and within territory scales of these hawks in Estonia [5] and in more southern parts of the species’ distribution [6–9].

**References**

1. Hakkarainen H, Mykrä S, Kurki S, Tornberg R, Jungell S. Competitive interactions among raptors in boreal forests. Oecologia. 2004;141: 420-424. doi: 10.1007/s00442-004-1656-6.

2. Byholm P, Nikula A, Kenttä J, Taivalmäki J. Interactions between habitat heterogeneity and food affect reproductive output in a top predator. J Anim Ecol. 2007;76: 392-401. doi: 10.1111/j.1365-2656.2007.01211.x.

3. Peltola A, Ihalainen A. Metsävarat (Forest resources; English summary: pp. 431–432). In: Ylitalo E, editor. Metsätilastollinen vuosikirja 2012 (Finnish Statistical Yearbook of Forestry). Vantaa: Finnish Forest Research Institute; 2012. pp. 37-78. Accessible: www.metla.fi/metinfo/tilasto/julkaisut/vsk/2012/.

4. Widén P. How, and why, is the goshawk (*Accipiter gentilis*) affected by modern forest management in Fennoscandia? J Raptor Res. 1997;31: 107-113.

5. Lõhmus A. Nest-tree and nest-stand characteristics of forest-dwelling raptors in east-central Estonia: Implications for forest management and conservation. Proc Estonian Acad Sci Biol Ecol. 2006;55: 31-50.

6. Gamauf A, Tebb G, Nemeth E. Honey buzzard *Pernis apivorus* nest-site selection in relation to habitat and the distribution of goshawks *Accipiter gentilis*. Ibis. 2013;155: 258-270. doi: 10.1111/ibi.12023.

7. Krüger O, Lindström J. Habitat heterogeneity affects population growth in goshawk *Accipiter gentilis*. J Anim Ecol. 2001;70: 173-181. doi: 10.1046/j.1365-2656.2001.00481.x.

8. Penteriani V, Faivre B. Breeding density and nest site selection in a goshawk *Accipiter gentilis* population of the Central Apennines (Abruzzo, Italy). Bird Study. 1997;44: 136-145. doi: 10.1080/00063659709461049.

9. Speiser R, Bosakowski T. Nest site selection by northern goshawks in northern New Jersey and southeastern New York. Condor. 1987;89: 387-394.
